# Survival of Ovarian Cancer Patients Is Independent of the Presence of DC and T Cell Subsets in Ascites

**DOI:** 10.3389/fimmu.2018.03156

**Published:** 2019-01-11

**Authors:** Christina Wefers, Tjitske Duiveman-de Boer, Refika Yigit, Petra L. M. Zusterzeel, Anne M. van Altena, Leon F. A. G. Massuger, I. Jolanda M. De Vries

**Affiliations:** ^1^Department of Tumor Immunology, Radboud Institute of Molecular Life Sciences, Radboud University Medical Centre (Radboudumc), Nijmegen, Netherlands; ^2^Department of Obstetrics and Gynecology, Radboud University Medical Centre (Radboudumc), Nijmegen, Netherlands

**Keywords:** ovarian cancer, ascites, immune environment, dendritic cells, T cells

## Abstract

Ascites is a prominent feature of ovarian cancer and could serve as liquid biopsy to assess the immune status of patients. Tumor-infiltrating T lymphocytes are correlated with improved survival in ovarian cancer. To investigate whether immune cells in ascites are associated with patient outcome, we analyzed the amount of dendritic cell (DC) and T cell subsets in ascites from ovarian cancer patients diagnosed with high-grade serous cancer (HGSC). Ascites was collected from 62 HGSC patients prior to chemotherapy. Clinicopathological, histological and follow-up data from patients were collected. Ascites-derived immune cells were isolated using density-gradient centrifugation. The presence of myeloid DCs (BDCA-1^+^, BDCA-3^+^, CD16^+^), pDCs (CD123^+^BDCA-2^+^), and T cells (CD4^+^, CD8^+^) was analyzed using flow cytometry. Complete cytoreduction, response to primary treatment and chemosensitivity were associated with improved patient outcome. In contrast, immune cells in ascites did not significantly correlate with patient survival. However, we observed a trend toward improved outcome for patients having low percentages of CD4^+^ T cells. Furthermore, we assessed the expression of co-stimulatory and co-inhibitory molecules on T cells and non-immune cells in 10 ascites samples. PD-1 was expressed by 30% of ascites-derived T cells and PD-L1 by 50% of non-immune cells. However, the percentage of DC and T cell subsets in ascites was not directly correlated to the survival of HGSC patients.

## Introduction

The majority of patients with epithelial ovarian cancer is diagnosed with advanced stage high-grade serous cancer (HGSC), with a poor 5-year overall survival rate of just 25% (1, 2). Standard treatment consists of cytoreductive surgery and platinum-based chemotherapy (3). Despite good initial response rates, about 70% of the patients relapse within 18 months (3–5). Unfortunately, over the last 30 years no significant improvement in survival is seen and, therefore, new treatment options are needed (6).

Ovarian cancer is an immunogenic disease and the tumor microenvironment is infiltrated by various immune cells that influence tumor progression and metastasis. The best studied immune cell type in solid tumors are T cells. CD8^+^ cytotoxic T cells kill tumor cells when they recognize an antigen that is presented by MHC class I. In ovarian cancer, tumor-infiltrating CD3^+^ and, more precisely, CD8^+^ T cells are associated with improved overall survival in advanced stage HGSC, as are high CD8/CD4 ratios (7–9).

In addition to T cells, DCs have been identified to infiltrate tumor tissue (10). DCs sample antigens and display them in an MHC-dependent context to T cells in the lymph node in order to initiate a potent anti-tumor T cell response. In blood, two main DC subsets exist that can be divided into myeloid DCs (mDCs) and plasmacytoid DCs (pDCs). Based on the expression of surface markers, mDCs can be further subdivided into BDCA-1^+^ and BDCA-3^+^ mDCs (11). There is much debate about the existence of a third, CD16 expressing mDC subset (12–14). Each DC subset has its own characteristics and function. Hence, the DCs present in the tumor microenvironment determine the nature of the generated T cell response (15). Tumor infiltrating DCs have been identified in many cancer types (16), but their correlation with patient outcome is unclear.

The tumor-suppressive microenvironment in ovarian cancer inhibits the function of effector T cells. Tumor cells down-regulate MHC molecules to escape detection by T cells (17). These tumor cells should be recognized and eliminated by natural killer (NK) cells (18). However, in ovarian cancer, NK cells are enriched for the less cytotoxic CD56^bright^CD16^−^ phenotype (19). In general, the link between infiltration of NK cells and patient survival is not yet clear. Furthermore, tumor cells attract and expand immunosuppressive cells, such as M2 macrophages and regulatory T cells (T_Regs_) (20). Depending on their activation, macrophages can acquire a M1 (classical activation) or M2 (alternative activation) phenotype. Whereas M1 macrophages exert anti-tumorigenic functions, M2 macrophages promote tumor progression (21). Therefore, a high M1/M2 ratio is associated with improved patient survival in ovarian cancer. In contrast, a higher density of M2 macrophages correlated with worse outcome (22). T_Regs_ inhibit the function of CD8^+^ T cells via cell-cell contact dependent mechanisms and by secreting inhibitory cytokines, such as IL-10, thereby limiting their tumor killing capacity (20). Tumor-infiltrating T_Regs_ are associated with worse patient outcome (23). The immunosuppressive tumor microenvironment also promotes the accumulation of immature DCs, which can induce tolerance and expand the T_Reg_ pool (24). Thus, boosting the pre-existing anti-tumor immune response by immunotherapy to overcome the immunosuppressive mechanisms should be a promising treatment strategy for ovarian cancer patients.

Immune infiltration into tumor tissue at baseline is an important predictor for patient's response to immunotherapy (25, 26). Acquisition of tumor material can only be achieved by invasive procedures. A prominent feature of advanced stage HGSC is fluid accumulation (ascites) within the peritoneal cavity. Ascites contains immune cells, such as macrophages, monocytes, granulocytes, and lymphocytes and could be seen as a reflection of the tumor microenvironment. The fluid can be obtained by drainage, which is an easier and safer procedure. Therefore, ascites could serve as liquid biopsy to assess the immune status of ovarian cancer patients (27, 28). However, so far it remains unclear whether immune cells in ascites can be correlated to clinical patient outcome. To investigate whether the different DC subsets correlate to T cell subsets, and whether these immune cells can be linked to patient survival and clinical characteristics, we analyzed the ascites of 62 HGSC patients.

## Methods

### Patient Material

This study was carried out in accordance with the recommendations of the “Dutch Code of Conduct for Responsible Use of Human Tissue,” established by the Federation of Dutch Medical Scientific Societies (Federa). Ascites is considered as waste material and, therefore, no written informed consent is needed. The protocol was approved by the “Commissie Mensgebonden Onderzoek” (CMO Arnhem-Nijmegen). Guidelines and regulations of the Radboudumc for collection of human material were followed. Ascites was collected from stage III or IV high-grade serous ovarian cancer patients before start of the treatment. The outcome of the cytoreductive surgery was defined as complete (no macroscopically visible residual tumor), optimal (residual tumor foci ≤ 1 cm) or suboptimal (residual tumor foci >1 cm). Staging was based on the International Federation of Gynecology and Obstetrics (FIGO) staging system. Data on adjuvant treatment, CA-125 serum levels, recurrence and last date of follow-up were collected. A good response to primary treatment was defined by a normalized serum CA-125 level, a normal physical examination and, if performed, a CT scan without evidence of recurrence. Patients with recurrence within 6 months after completion of chemotherapy were classified as having chemoresistant tumors. Progression-free survival (PFS) was defined as period of time (months) between last course of chemotherapy and recurrence. Overall survival (OS) was defined as the period of time (months) between the last course of chemotherapy and death or date of last follow-up.

### Isolation of Immune Cells From Ascites

Immune cells were isolated as described elsewhere (29). In brief, ascites was filtered over a 100 μm cell strainer (352360, Corning, Falcon®) and centrifuged for 15 min at 1,500 rpm and 4°C. The cell pellet was resuspended in phosphate buffered saline (PBS). Mononuclear cells were isolated using gradient centrifugation with Lymphoprep^TM^ (1114544, Axis-Shield). Isolated immune cells were cryopreserved in a dimethyl sulfoxide (DMSO, 06800001, WAK-Chemie)-containing medium.

### Flow Cytometry

Ascites-derived mononuclear cells were blocked for 10 min with 50 μl PBA/2% FcR blocking reagent (130-090-532, Miltenyi). Subsequently, cells were incubated for 30 min with directly conjugated antibodies. The cells were washed with PBA and analyzed. Different panels were used for immune cell phenotyping. T cells were identified using antibodies against CD3, CD4, and CD8. mDCs were identified using antibodies against CD45, CD14, CD16, CD19, BDCA-1 (CD1c), and BDCA-3 (CD141). For pDCs, antibodies against CD123 and BDCA-2 (CD303) were used. Immune checkpoint expression was analyzed on CD3+ T cells and CD45- cells.

### Antibodies

The following antibodies were used: CD3-FITC (1:25, 555339, BD Pharmingen), CD3-BV510 (1:25, 563083, BD), CD4-APC-Cy7 (1:20, 300518, BioLegend), CD8-PerCP (1:20, 345774, BD), CD14-APC-Cy7 (1:20, 557831, BD Pharmingen), CD16-PE-Cy7 (1:200, 335823, BD), CD19-PerCP (1:2.5, 345778, BD), CD40-PE (1:10, IM19360U, Beckman), CD40-L-PE (1:10, IM2216U, Beckman), CD45-V450 (1:50, 560367, BD), CD45-FITC (1:10, 130-080-202, Miltenyi Biotec), CD80-PE-Cy7 (1:50, 561135, BD), CD86-PE (1:10, 555658, BD) CD123-FITC (1:10, 130-090-897, Miltenyi Biotec), BDCA-1-PE (1:10, 130-090-508, Miltenyi Biotec), BDCA-2-APC (1:10, 130-090-905, Miltenyi Biotec), BDCA-3-APC (1:10, 130-090-907, Miltenyi Biotec), CTLA-4-PE (1:5, 555853, BD), HLA-DR-BV510 (1:25, 563083, BD), ICOS-PerCP-Cy5.5 (1:10, 562833, BD), ICOS-L-PerCP (1:5, FAB165C, R&D Systems), PD-1-PerCP-Cy5.5 (1:10, 561273, BD), PD-L1-PE-Cy7 (1:10, 558017, BD).

### Statistical Analysis

Comparisons between DC and T cell subsets were made using Pearson's *r*-test on Log-transformed data. The Mann-Whitney *U*-test was used for comparisons between clinicopathological characteristics and DC and T cell subset percentages. Correlations between immune cell populations and clinical characteristics were done using univariate Cox regression analysis. Correlations between immune cell populations and survival were analyzed using Cox regression and Kaplan-Meier survival curves (log-rank test). All *p-*values were considered significant when *p* < 0.05. SPSS 22.0 software was used for statistical analyses.

## Results

### Patient Characteristics

Ascites from 62 ovarian cancer patients was collected prior to any chemotherapy treatment via ascites drainage or during primary surgery (Table 1). All patients were diagnosed with HGSC. Out of 62 patients, 52 were diagnosed with FIGO stage III and 10 with stage IV disease. The median age at diagnosis was 64 years (range 42–80 years). One patient was treated with chemotherapy only and one patient underwent cytoreductive surgery only. Twenty two patients underwent a primary debulking, followed by six courses of adjuvant chemotherapy. The remaining 39 patients received three courses of neo-adjuvant chemotherapy, followed by interval debulking and another three courses of adjuvant chemotherapy. Complete or optimal (≤ 1 cm residual tumor foci) cytoreduction was achieved in 26 and 28 patients, respectively, whereas 7 patients had a suboptimal (>1 cm residual tumor foci) debulking. The majority of patients received combination chemotherapy, consisting of taxol and platinum (cisplatin, carboplatin), and six patients received carboplatin monotherapy. A good response to primary treatment was observed in 38 patients. Median PFS and OS was 7 months (range 0–95) and 21 months (range 1–99), respectively.

**Table 1:** Clinicopathological characteristics of high-grade serous ovarian cancer patients.

	***N* (62)**	**%**
Median age (range)	64	(42–80)
**FIGO STAGE**		
III	52	84
IV	10	16
**SURGERY**		
Primary cytoreduction	22	35
Secondary cytoreduction	39	63
No cytoreduction	1	2
**OUTCOME SURGERY**		
Complete	26	42
Optimal (≤ 1 cm)	28	46
Suboptimal (>1 cm)	7	12
**CHEMOTHERAPY**		
Taxol/Platinum	56	90
Non-taxol	5	8
No chemotherapy	1	2
**RESPONSE TO PRIMARY TREATMENT**		
Responder	38	63
Non-responder	22	37

*IGO, Fédération Internationale de Gynécologie Obstétrique*.

### mDCs, pDCs, and T Cells Are Present in Ascites

**Figure 1:**
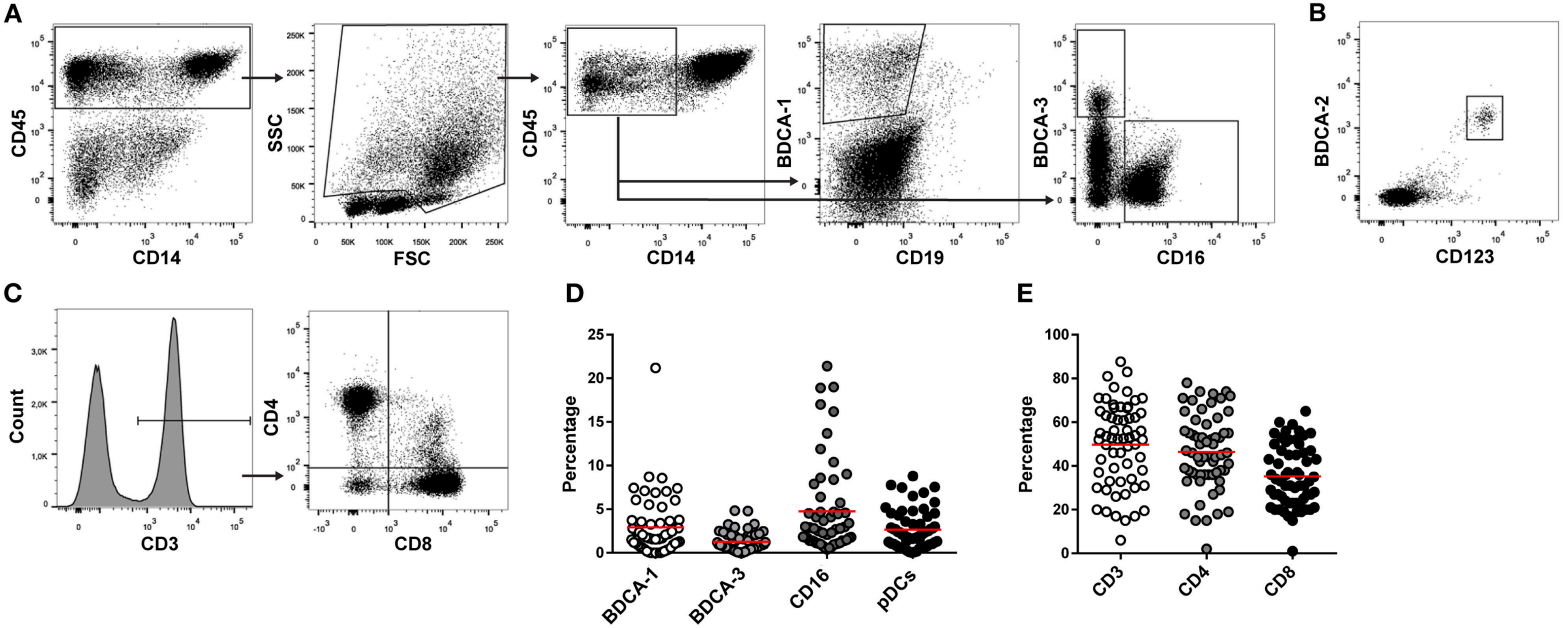
T cell and DC subsets in ascites. DC en T cells subsets were measured in ascites of 62 high-grade serous ovarian cancer patients using flowcytometry. **(A)** Gating strategy for myeloid dendritic cells. Lymphocytes were excluded based on FSC/SSC. BDCA-1^+^, BDCA-3^+^, and CD16^+^ mDCs were gated in the CD45^+^CD14^−^ cell population. **(B)** pDCs were identified based on co-expression of BDCA-2 and CD123. **(C)** T cell gating strategy. CD4^+^ and CD8^+^ T cells were gated in the CD3^+^ lymphocyte population. **(D)** Percentage of DC subsets in ascites. **(E)** Percentage of T cells in ascites. Red line indicates mean.

DC and T cell subsets in ascites were analyzed using flow cytometry. Due to limited cell numbers in some samples, mDCs were measured in 56 samples and pDCs and T cells in 62 ascites samples. BDCA-1^+^, BDCA-3^+^, and CD16^+^ mDCs were analyzed in the CD45^+^CD14^−^ cell population (Table 2, Figure 1A). pDCs were identified by co-expression of CD123 and BDCA-2 (Figure 1B). BDCA-1^+^ and BDCA-3^+^ mDCs were detected in 91% (51/56) and 96% (54/56) of ascites samples, respectively. CD16^+^ mDCs and pDCs were present in 100% (56/56 and 62/62) of patient samples. The median percentage of the different DC subsets in ascites was 1.8% for BDCA-1^+^ mDCs (range 0.0–21.2%), 0.9% for BDCA-3^+^ mDCs (range 0.0–4.9%), 2.8% for CD16^+^ mDCs (range 0.4–21.4%), and 2.1% for pDCs (range 0.1–8.8%) (Figure 1D). T cells were detected in 100% (62/62) of ascites samples. The median percentage of T cell subsets in the CD3^+^ cell population was 45.5% for CD4^+^ T cells (range 2.0–78.0%), containing T helper cells and T_Regs_, and 33.0% for CD8^+^ cytotoxic T cells (range 1.0–65.0%) (Figures 1C,E). The correlation between DC and T cell subsets was investigated by calculating the Pearson correlation coefficient. There was no correlation between BDCA-1^+^, BDCA-3^+^, and CD16^+^ mDCs and the different T cell subsets. In contrast, percentages of pDCs positively correlated with higher percentages of CD4^+^ T cells (*r* = 0.348, *p* = 0.048).

**Table 2:** Markers used for the identification of mDCs, pDCs and T cells by flowcytometry.

**Cell type**	**Markers**	**Function**
BDCA-1^+^ mDCs	CD45^+^CD14^−^BDCA-1^+^	Directing polarization of CD4^+^ T helper cells to Th1, Th2 or Th17 phenotype
BDCA-3^+^ mDCs	CD45^+^CD14^−^BDCA-3^+^	Polarization of CD4^+^ T helper cells to Th1 phenotype; Cross-presentation of antigens to CD8^+^ T cells
CD16^+^ mDCs	CD45^+^CD14^−^CD16^+^	Antibody dependent cellular cytotoxicity;
pDCs	CD123^+^BDCA-2^+^	Secretion of type I interferons; induction of T regulatory cells
CD4^+^ T cells	CD3^+^CD4^+^	CD4^+^ T helper cells aid to initiate a proper immune response; CD4^+^ T regulatory cells dampen the immune response
CD8^+^ T cells	CD3^+^CD8^+^	Cytotoxic T cells; kill infected or cancerous cells

### PFS and OS Are Independent of the Percentage of DC and T Cell Subsets in Ascites

**Figure 2:**
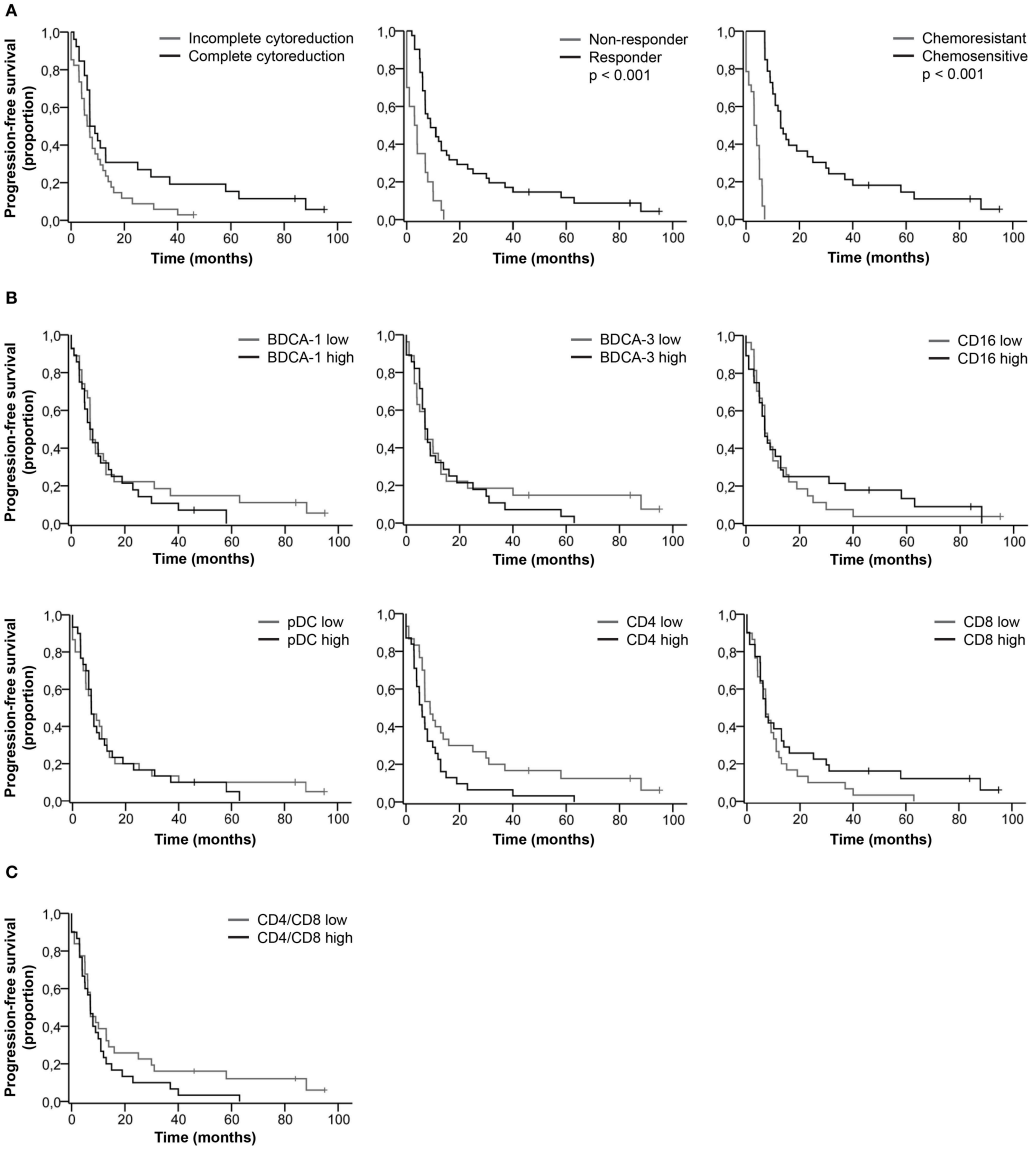
Kaplan-Meier curves for progression-free survival of HGSC patients. **(A)** Progression-free survival curves for clinical characteristics. **(B)** Progression-free survival for patients stratified as having low or high percentages of immune cells in ascites. Cut-off values based on median. BDCA-1: 1.8%; BDCA-3: 0.9%; CD16: 2.8%; pDC: 2.1%; CD4: 45.5%; CD8: 33.0%; **(C)** Progression-free survival for patients stratified as having low or high CD4/CD8 ratios. Cut-off at 1.3, based on population median. *P*-values for significant differences are given. *P-values* < 0.05 were considered significant.

**Figure 3:**
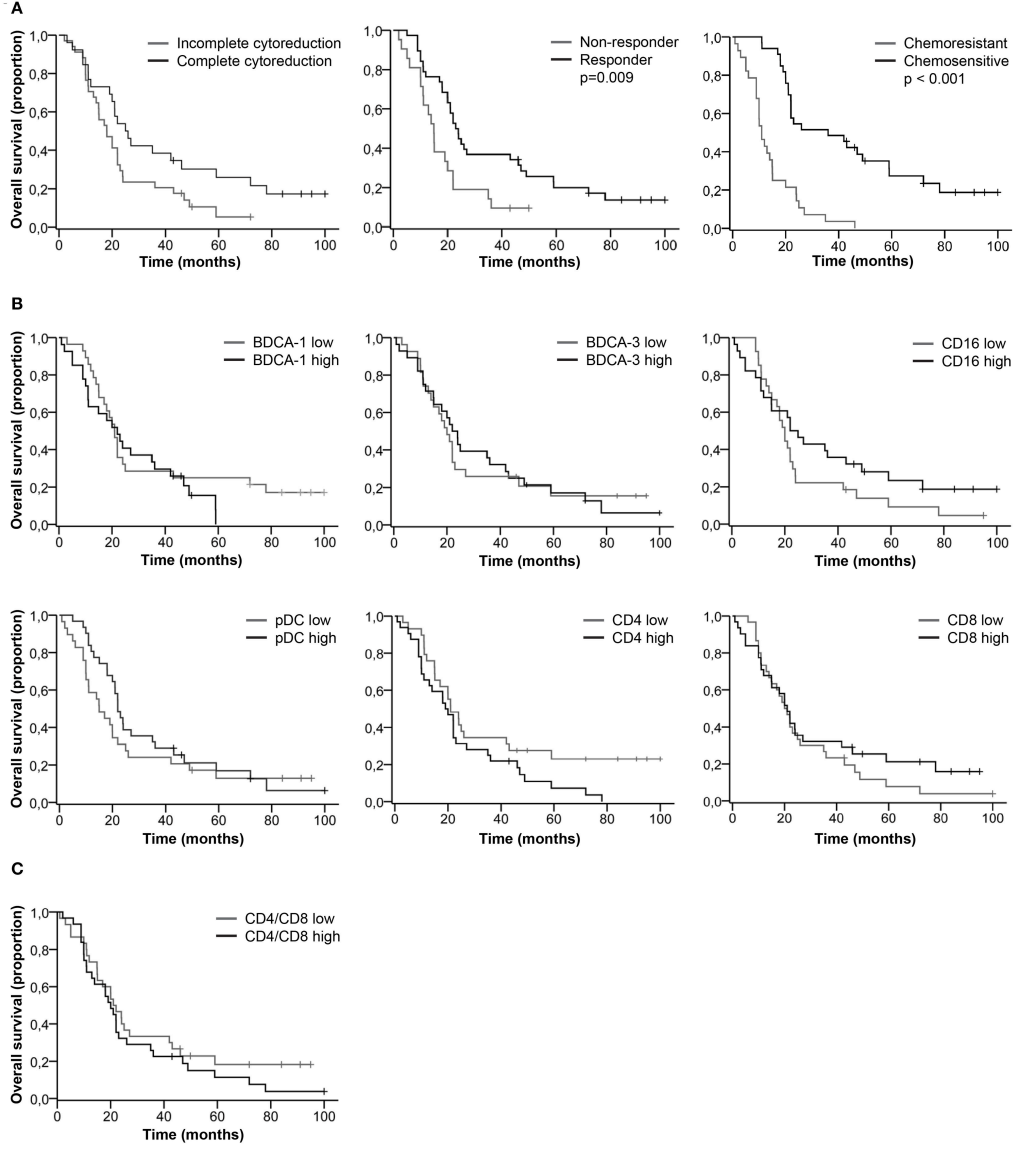
Kaplan-Meier curves for overall survival of HGSC patients. **(A)** Overall survival curves for clinical characteristics. **(B)** Overall survival for patients stratified as having low or high percentages of immune cells in ascites. Cut-off values based on median. BDCA-1: 1.8%; BDCA-3: 0.9%; CD16: 2.8%; pDC: 2.1%; CD4: 45.5%; CD8: 33.0%; **(C)** Overall survival for patients stratified as having low or high CD4/CD8 ratios. Cut-off at 1.3, based on population median. *P*-values for significant differences are given. *P-values* < 0.05 were considered significant.

We did not find a significant difference in the percentage of DC and T cell subsets between complete vs. incomplete cytoreduction, responders vs. non-responders, and chemosensitive vs. chemoresistant tumors (Table S1). Clinical characteristics significantly correlated with patient outcome. Patients with complete cytoreduction, a good response to primary treatment and chemo sensitive primary tumors had improved PFS (Figure 2A) and OS (Figure 3A, Table S2). Furthermore, based on the median, patients were stratified as having low or high percentages of immune cells. There was no significant difference in PFS (Figures 2B,C) and OS (Figures 3B,C) for patients with low or high percentages of DC and T cell subsets in ascites, although we observed a trend toward improved PFS and OS for patients having low percentages of CD4^+^ T cells in ascites.

### Trend Toward Improved PFS and OS for Patients With High Percentages of CD16^+^ mDCs and Low Percentages of CD4^+^ T Cells

Even though the percentage of DC and T cell subsets did not significantly correlate with patient outcome, long PFS and OS were more likely to occur in patients with a low percentage of CD4^+^ T cells and a high percentage of CD16^+^ mDCs (Table S3). For patients with a low percentage of CD4^+^ T cells, the PFS rate at 18 months was 30.0% and the OS rate at 60 months was 17.0%, in contrast to 13.0 and 7.0%, respectively, for patients with a high percentage of CD4^+^ T cells. Furthermore, 26.0% of patients with a high percentage of CD16^+^ mDCs were free of recurrence after 18 months and 19.0% alive at 60 months, whereas the probability of PFS and OS for patients with a low percentage of CD16^+^ mDCs was 19.0 and 7.0%, respectively.

### Ascites-Derived T Cells Are Positive for the Inhibitory Checkpoint PD-1

**Figure 4:**
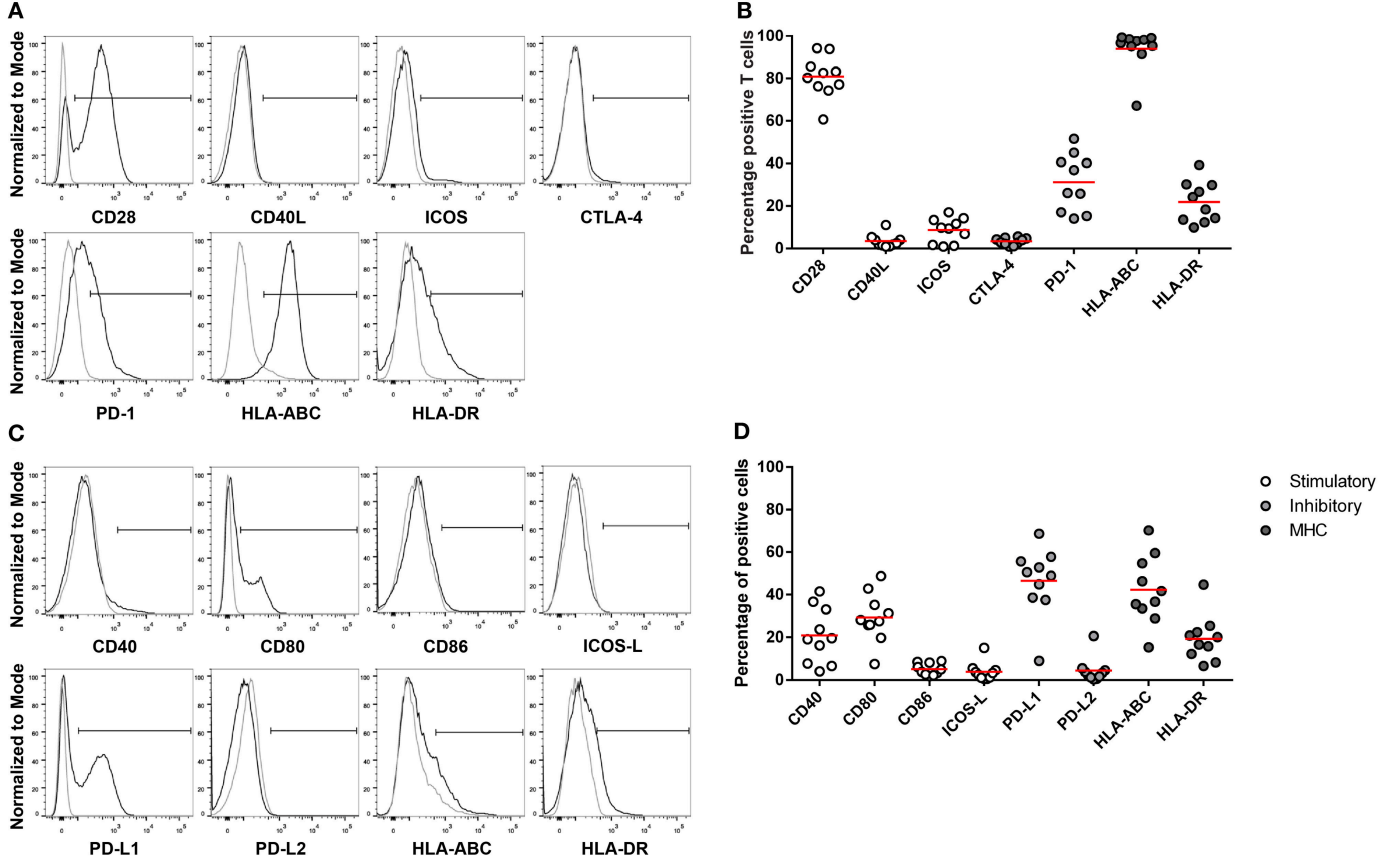
Expression of MHC, co-stimulatory and co-inhibitory molecules on CD3^+^ T cells and non-immune cells in ascites. Flow cytometry gating of immune checkpoint and MHC on **(A)** CD3^+^ positive cells and **(C)** CD45^−^ cells. Gray line represents control; black line represents CD3^+^ T cells or CD45^−^ cells. **(B)** CD3^+^ T cells and **(D)** CD45^−^ cells positive for co-stimulatory, co-inhibitory, and MHC molecules. Red lines indicate mean.

Since the percentage of CD4^+^ and CD8^+^ T cells was not directly correlated to clinical characteristics of HGSC patients, we investigated the activation status of ascites-derived T cells. The expression of immune checkpoint markers was investigated in 10 ascites samples, being 6 responders and 4 non-responders, of which enough cells were available for further analysis. Checkpoint marker expression was analyzed on CD3^+^ T cells (Figures 4A,B) and on the CD45^−^ cell population (Figures 4C,D).

The co-stimulatory molecule CD28 was detected on 81.6% of T cells, whereas CD40L and ICOS were expressed on a minority of cells, 4.8 and 8.1%, respectively. About 21.4% of the T cells were positive for the activation marker HLA-DR. The co-inhibitory molecule CTLA-4 was barely present on T cells. In contrast, the mean of PD-1 expressing cells was 30.7%, ranging from 14 to 51.5% in the ascites samples.

We also analyzed the expression of the corresponding ligands and receptors on the CD45^−^ cell population in ascites. The co-stimulatory molecule CD28 competes with the co-inhibitory molecule CTLA-4 for binding to CD80 and CD86. About 32.0% of non-immune cells expressed the co-stimulatory molecule CD80, whereas CD86 is only expressed by 5.4%. The ligand for the ICOS receptor, ICOS-L, was present on 3.8% of non-immune cells. CD40, which binds to CD40L, was detected on 22.4.0% and HLA-ABC, important for antigen presentation to CD8^+^ T cells, was detected on 44.1% of CD45^−^ cells. On average, 48.8% of non-immune cells were positive for the ligand for the co-inhibitory molecule PD-1, PD-L1, ranging from 9.1 to 72.3%.

## Discussion

The aim of this study was to investigate whether the main DC and T cell subsets in ascites are correlated to clinical characteristics or patient outcome. We detected DCs and T cells in a majority of ascites samples. However, survival of ovarian cancer patients was largely independent of the amount of these cells in ascites.

DC subsets were analyzed in the CD45^+^CD14^−^ cell population. The rationale to study DCs is that each subset has its own characteristics and functions. Whereas, BDCA-1^+^ mDCs direct the polarization of CD4^+^ T helper cells, BDCA-3^+^ DCs are potent at cross-presenting antigens to CD8^+^ T cells and, therefore, have an important role in inducing T cell responses against tumor cells (11). There is much debate about whether CD16^+^ mDCs are actual DCs or non-classical monocytes. Nevertheless, these cells are potent in antibody-dependent cellular cytotoxicity and induce T cell proliferation and we observed a trend toward improved survival in patients with high percentages of CD16^+^ mDCs (13). However, we did not find a correlation between mDC subsets and CD4^+^ or CD8^+^ T cells. pDCs have a strong pro-inflammatory function by secreting type I IFNs in response to viral infections (11). On the other hand, they produce indoleamine 2,3-dioxygenase (IDO) and induce T_Regs_, thereby, dampening the immune system (11, 20). We found a positive correlation between pDCs and CD4^+^ T cells in ascites of HGSC patients. We additionally observed a trend toward improved PFS and OS in patients with low percentages of CD4^+^ T cells. Patients with a high percentage of CD4^+^ T cells had worse clinical outcome, pointing toward a regulatory phenotype of these cells. However, there was no significant link between the percentage of DC and T cell subsets in ascites and survival of HGSC patients. In contrast, clinical characteristics, such as complete cytoreduction, good response to primary treatment, and chemosensitivity correlated to improved patient outcome.

Ascites is an immunosuppressive milieu, which could explain why immune cells in ascites are not correlated to patient outcome. We as well as others, showed that ascites interferes with T cell function. This inhibition is mediated by various factors, such as lipids, chemokines, and by the presence of regulatory immune cells in ascites fluid (30–33). For example, Landskron et al. characterized the T cell population in ascites and peripheral blood of ovarian cancer patients. They demonstrated that activated cytokine-secreting T_Regs_ were enriched in ascites (34). Furthermore, ascites contains high levels of IL-10, which can stimulate the expansion of the T_Reg_ population and inhibits CD8^+^ T cell function (35). Additionally, ascites affects the polarization of macrophages. IL-6 and IL-10 in ascites promote the differentiation of macrophages into the immunosuppressive M2 phenotype, which stimulates tumor cell proliferation and suppresses the effector functions of cytotoxic T cells (36, 37). We also detected that 30% of the T cells in ovarian cancer ascites were positive for PD-1. The ligand PD-L1 is present on 50% of the CD45^−^ population, which contains tumor cells. The PD-1/PD-L1 axis could be a mechanism that interferes with the cytotoxic activity of CD8^+^ T cells in ascites. However, it remains to be elucidated whether PD-1 expression is a marker for exhaustion or activation. Next to T cells, ascites fluid may also inhibit the function of DCs. Recently, ascites fluid was shown to interfere with the activation of *in vitro* generated monocyte-derived DCs. Ascites reduced the expression of the co-stimulatory molecule CD86 and decreased the T cell stimulatory capacity (38). Hence, ovarian cancer ascites may effectively limit the anti-tumor immune response and, therefore, ascites-derived immune cells might not correlate with patient outcome. In the future, it might be important to assess the activation status of DC and T cell subsets in ovarian cancer ascites.

So far, only a few research groups have investigated the effect of immune cells in ascites on the outcome of ovarian cancer patients. These studies found contradicting results and are partly in contrast to our own findings. For example, Giuntoli et al. showed that the ratio of CD4/CD8 T cells in ovarian cancer ascites is correlated to patient outcome. They demonstrated that patients with low CD4/8 ratios had improved survival (39), a finding we could not confirm. In contrast, Lieber et al. found no correlation of CD4^+^ or CD8^+^ T cells with patient outcome, but identified memory T cells to confer survival advantage in HGSC patients (40). Although not statistically significant, we observed a trend toward improved outcome of patients with low percentages of CD4^+^ T cells. The group of Bamias et al. found that CD3^+^CD56^+^ cells in ascites were associated with platinum resistance (41), whereas we did not observe a correlation of DC or T cell subsets with platinum resistance. Hoogstad-van Evert et al. detected no association of CD3^+^ T cells with patient outcome, but found NK cells in ovarian cancer ascites to be correlated with improved survival (42). These studies used a limited number of samples or were composed of a heterogeneous patient population, including different epithelial ovarian cancer subtypes and material from before and after therapy. In this study, we tested the ascites of a well-defined group of patients, all being chemotherapy naïve and with diagnosed advanced-stage HGSC.

In summary, we showed that DC and T cell subsets in ascites, in contrast to tumor infiltrating T cells, did not correlate with clinical characteristics or survival of HGSC patients. Future research needs to directly compare the immune infiltration in ascites and primary tumor tissue to draw conclusions on the immune environment in these two compartments. Furthermore, research on ascites would benefit from lager patient cohorts, as this would allow for more advanced statistical analysis. This requires the systematic biobanking of ascites, which is currently not done. Finally, homogenous patient cohorts are crucial to reliably correlate immune cells in ascites with patient outcome. This study serves as starting point for future research investigating the immune infiltrate in ovarian cancer ascites.

## Author Contributions

CW, TD-dB, and RY performed and analyzed immunological experiments. PZ, LM, and AvA collected ascites and patient data. CW wrote the manuscript. LM and ID designed and supervised the study. All authors read and approved the final version of the manuscript.

### Conflict of Interest Statement

The authors declare that the research was conducted in the absence of any commercial or financial relationships that could be construed as a potential conflict of interest.
