# Supplementary material for: Survival of Ovarian Cancer Patients Is Independent of the Presence of DC and T Cell Subsets in Ascites
Source: Front Immunol. 2019 Jan 11;9:3156. doi: 10.3389/fimmu.2018.03156 (PMC6336918; doi:10.3389/fimmu.2018.03156)
Supplement: Supplementary file 1 [file Data_Sheet_1.docx]

Supplementary Material

Immune environment in ovarian cancer ascites

Christina Wefers, Tjitske Duiveman-de Boer, Refika Yigit, Petra L.M. Zusterzeel, Anne van Altena, Leon F.A.G. Massuger, I. Jolanda M. de Vries^*^

*** Correspondence:** I. Jolanda M. de Vries, [Jolanda.deVries@radboudumc.nl](mailto:Jolanda.deVries@radboudumc.nl)

**Supplementary Table S1.** Comparison of the percentage DC and T cell subsets with clinicopathological characteristics.

|  | **BDCA-1** | **BDCA-3** | **CD16** | **pDCs** | **CD4** | **CD8** | **CD4/CD8** |
| --- | --- | --- | --- | --- | --- | --- | --- |
| **Cytoreduction**  *Complete*  *Not complete* | 1.2  2.2 | 0.9  0.7 | 3.2  2.1 | 2.1  2.1 | 42.0  53.0 | 36.0  28.5 | 1.2  1.6 |
| **CaA-125 after treatment**  *Normal*  *Elevated* | 1.5  2.9 | 0.9  0.9 | 3.6  4.0 | 2.3  2.5 | 43.0  53.5 | 35.5  28.0 | 1.2  1.9 |
| **Response to chemo**  *Chemosensitive*  *Chemoresistant* | 1.7  2.5 | 0.9  0.9 | 2.2  4.4 | 2.5  2.0 | 43.0  46.5 | 32.0  33.5 | 1.4  1.3 |
| **Response to treatment**  *Responder*  *Non-responder* | 1.5  2.1 | 0.9  0.7 | 2.8  2.6 | 2.4  2.0 | 44.0  52.8 | 35.5  30.0 | 1.2  1.8 |

Comparison using Mann-Whitney U analysis.

**Supplementary Table S2.** Univariate Cox regression analysis on progression-free and overall survival.

|  |  |  | **Progression- free survival** | | | **Overall survival** | | | |  |
| --- | --- | --- | --- | --- | --- | --- | --- | --- | --- | --- |
|  | **Variables** | ***n*** | **HR** | **95% CI** | ***p* value** | | **HR** | **95% CI** | ***p* value** | |
| **Clinical characteristics** | ***Complete cytoreduction*** |  |  |  |  | |  |  |  | |
|  | *Complete* | 26 | 1 |  |  | | 1 |  |  | |
|  | *Not complete* | 35 | 1.70 | 0.98-2.96 | 0.061 | | 1.76 | 0.93-3.14 | 0.053 | |
|  | ***Response to chemotherapy*** |  |  |  |  | |  |  |  | |
|  | *Chemo sensitive* | 33 | 1 |  |  | | 1 |  |  | |
|  | *Chemo resistant* | 28 | 3.56 | 2.98-10.32 | 0.002 | | 4.80 | 2.60-8.84 | <0.001 | |
|  | ***Response to treatment*** |  |  |  |  | |  |  |  | |
|  | *Responder* | 38 | 1 |  |  | | 1 |  |  | |
|  | *Non-responder* | 22 | 2.76 | 1.56-4.90 | 0.001 | | 2.16 | 1.19-3.93 | 0.011 | |
| **DC subsets** | ***BDCA-1^+^ mDC*** |  |  |  |  | |  |  |  | |
|  | *Low (< 1.7)* | 28 | 1 |  |  | | 1 |  |  | |
|  | *High (≥ 1.7)* | 28 | 1.27 | 0.72-2.22 | 0.411 | | 1.33 | 0.73-2.41 | 0.349 | |
|  | ***BDCA-3^+^ mDC*** |  |  |  |  | |  |  |  | |
|  | *Low (< 0.9)* | 28 | 1 |  |  | | 1 |  |  | |
|  | *High (≥ 0.9)* | 28 | 1.21 | 0.69-2.11 | 0.498 | | 0.96 | 0.54-1.70 | 0.885 | |
|  | ***CD16^+^ mDC*** |  |  |  |  | |  |  |  | |
|  | *Low (< 2.8)* | 28 | 1 |  |  | | 1 |  |  | |
|  | *High (≥ 2.8)* | 28 | 0.87 | 0.50-1.53 | 0.644 | | 0.68 | 0.38-1.21 | 0.190 | |
|  | ***pDC*** |  |  |  |  | |  |  |  | |
|  | *Low (< 2.1)* | 31 | 1 |  |  | | 1 |  |  | |
|  | *High (≥ 2.1)* | 31 | 1.09 | 0.65-1.85 | 0.737 | | 0.76 | 0.44-1.31 | 0.318 | |
| **T cell subsets** | ***CD4^+^ T cell*** |  |  |  |  | |  |  |  | |
|  | *Low (< 45.5)* | 31 | 1 |  |  | | 1 |  |  | |
|  | *High (≥ 45.5)* | 31 | 1.83 | 1.07-3.11 | 0.182 | | 1.66 | 0.96-2.88 | 0.189 | |
|  | ***CD8^+^ T cell*** |  |  |  |  | |  |  |  | |
|  | *Low (< 33)* | 30 | 1 |  |  | | 1 |  |  | |
|  | *High (≥ 33)* | 32 | 0.72 | 0.42-1.21 | 0.215 | | 0.78 | 0.45-1.34 | 0.369 | |
|  | ***CD4/CD8 T cell ratio*** |  |  |  |  | |  |  |  | |
|  | *Low (< 1.3)* | 31 | 1 |  |  | | 1 |  |  | |
|  | *High (≥ 1.3)* | 31 | 1.43 | 0.85-2.41 | 0.183 | | 1.34 | 0.78-2.30 | 0.294 | |

HR: hazard ratio; CI: confidence interval; mDC: myeloid dendritic cell; pDC: plasmacytoid dendritic cell.

**Supplementary Table S3.** PFS and OS rate (%) for patients with a low or high percentage of DC and T cell subsets.

|  | **BDCA-1** | | **BDCA-3** | | **CD16** | | **pDCs** | | **CD4** | | **CD8** | |
| --- | --- | --- | --- | --- | --- | --- | --- | --- | --- | --- | --- | --- |
|  | **Low** | **High** | **Low** | **High** | **Low** | **High** | **Low** | **High** | **Low** | **High** | **Low** | **High** |
| **PFS** |  |  |  |  |  |  |  |  |  |  |  |  |
| ≥ 12 months | 30 | 33 | 33 | 30 | 26 | 37 | 23 | 31 | 40 | 23 | 23 | 37 |
| ≥ 18 months | 22 | 22 | 22 | 19 | 19 | 26 | 17 | 24 | 30 | 13 | 17 | 23 |
| ≥ 24 months | 22 | 15 | 19 | 15 | 11 | 26 | 17 | 17 | 30 | 7 | 10 | 23 |
| **OS** |  |  |  |  |  |  |  |  |  |  |  |  |
| ≥ 48 months | 26 | 15 | 15 | 26 | 11 | 30 | 20 | 17 | 23 | 13 | 13 | 23 |
| ≥ 60 months | 26 | 0 | 11 | 15 | 7 | 19 | 10 | 14 | 17 | 7 | 7 | 17 |
| ≥ 72 months | 19 | 0 | 11 | 7 | 7 | 11 | 10 | 7 | 13 | 3 | 3 | 13 |

PFS: Progression-free survival; OS: Overall survival.
